# Urinary proteomic profiling to identify early biomarkers of non-diabetic chronic kidney disease

**DOI:** 10.1093/ckj/sfaf313

**Published:** 2025-10-06

**Authors:** Henry H L Wu, Naveen Kumar Parthiban, Muhammad A Zenaidee, Ignatius Pang, Yunqi Wu, Seong Beom Ahn, Robert J Walker, Carol A Pollock, Sonia Saad

**Affiliations:** Renal Research, Kolling Institute of Medical Research, Royal North Shore Hospital & The University of Sydney, Sydney, Australia; Department of Renal Medicine, Royal North Shore Hospital, Northern Sydney Local Health District, Sydney, Australia; Renal Research, Kolling Institute of Medical Research, Royal North Shore Hospital & The University of Sydney, Sydney, Australia; Mass Spectrometry Department, Australian Proteome Analysis Facility, Macquarie University, Sydney, Australia; Mass Spectrometry Department, Australian Proteome Analysis Facility, Macquarie University, Sydney, Australia; Mass Spectrometry Department, Australian Proteome Analysis Facility, Macquarie University, Sydney, Australia; Macquarie Medical School, Macquarie University, Sydney, Australia; Department of Medicine, University of Otago, Dunedin, New Zealand; Renal Research, Kolling Institute of Medical Research, Royal North Shore Hospital & The University of Sydney, Sydney, Australia; Department of Renal Medicine, Royal North Shore Hospital, Northern Sydney Local Health District, Sydney, Australia; Renal Research, Kolling Institute of Medical Research, Royal North Shore Hospital & The University of Sydney, Sydney, Australia

To the Editor,

Proteinuria is established as a key indicator of chronic kidney disease (CKD) progression and severity. The kidneys excrete hundreds of different proteins. While the profiles and utility of urinary proteomic biomarkers in identifying diabetic kidney disease and to monitor longitudinal progression of CKD was well-investigated [1, 2], there is a paucity of published data relating to significant urinary proteomic biomarkers specifically within the context of identifying early-stage non-diabetic CKD. The non-diabetic population living with early-stage CKD is a prevalent cohort at risk of adverse outcomes, and early disease identification followed by treatment initiation is important for disease optimization [3]. In this pilot study, we conducted urinary proteomic profiling analyses to identify potential biomarkers of early-stage CKD in non-diabetic individuals.

This was a cross-sectional study in which urinary proteomic profiling was performed for 20 adult male non-diabetic patients who underwent kidney biopsy. CKD status was determined as per interstitial fibrosis and tubular atrophy (IFTA) grading. There were 10 patients with IFTA 0%–10% (no CKD) and 10 patients with IFTA 10%–25% (early-stage CKD) (Table 1). The two groups were statistically similar in age, a median age 67 years [interquartile range (IQR) 41–71 years] in the no CKD group versus 62 years (IQR 57–68 years) in the early-stage CKD group, *P* = .880; sex, all 20 patients were male; and estimated glomerular filtration rate (eGFR), a median eGFR >90 ml/min/1.73 m^2^ (IQR >90–>90 ml/min/1.73 m^2^) in the no CKD group versus 88 ml/min/1.73 m^2^ (IQR 86–90 ml/min/1.73 m^2^) in the early-stage CKD group, *P* = .246. In the early-stage CKD group, there were seven patients with micro- or macroalbuminuria. Urine samples were collected immediately before kidney biopsy and prepared for data independent acquisition liquid chromatography-mass spectrometry analysis. Database searches for data independent acquisition data and statistical differential abundances analysis of the searched data by RStudio (v.4.4.2) were completed, followed by data filtering and normalization.

**Table 1:** Information on age, eGFR, albuminuria status, co-morbidities, treatments received at the timepoint of kidney biopsy, and diagnosis following kidney biopsy for the 20 study patients.

Patient ID	Age (years)	eGFR (ml/min/1.73 m^2^)	Albuminuria status	Co-morbidities	Treatments received at the timepoint of kidney biopsy	Diagnosis following histological evaluation of kidney biopsy tissue
IFTA 0%–10% (*n* = 10)						
1	31	>90	Nil	Asthma	Budesonide, Salbutamol	Nil kidney disease—Minimal glomerular sclerosis and IFTA noted
2	73	>90	Nil	Osteoarthritis	Paracetamol, Voltaren emulgel	Nil kidney disease—very minimal glomerular sclerosis noted
3	71	>90	Nil	Hypercholesterolemia	Atorvastatin	Nil kidney disease
4	48	>90	Nil	Hypertension Trigeminal neuralgia	Indapamide, Losartan, Carbamazepine	Nil kidney disease—very minimal hypertensive nephrosclerotic changes noted
5	68	>90	Nil	Nil	Nil	Nil kidney disease
6	77	>90	Nil	Hypercholesterolemia	Simvastatin	Nil kidney disease –minimal glomerular sclerosis and IFTA noted
7	41	>90	Nil	Nil	Nil	Nil kidney disease
8	66	>90	Nil	Rheumatoid arthritis	Methotrexate, Folic acid, Paracetamol, Ibuprofen	Nil kidney disease
9	73	>90	Nil	Hypercholesterolemia	Rosuvastatin	Nil kidney disease—minimal glomerular sclerosis and IFTA noted
10	68	>90	Nil	Hypertension	Indapamide	Nil kidney disease—very minimal hypertensive nephrosclerotic changes noted
**IFTA 10–25% (n = 10)**						
11	79	>90	Micro	Osteoarthritis	Paracetamol, Voltaren emulgel	Non-steroidal anti-inflammatory drug-induced kidney tubulointerstitial fibrosis
12	68	88	Micro	Hypertension	Indapamide, Losartan, Prazosin	Hypertensive nephropathy
13	64	>90	Micro	Hypertension Hypercholesterolemia	Indapamide, Losartan, Rosuvastatin	Hypertensive nephropathy
14	61	>90	Nil	Nil	Nil	Immunoglobulin A nephropathy
15	48	88	Micro	Hypertension	Indapamide, Losartan	Hypertensive nephropathy
16	67	87	Micro	Hypertension	Indapamide, Losartan	Immunoglobulin A nephropathy
17	57	>90	Nil	Hypercholesterolemia	Rosuvastatin	Early CKD changes due to high cholesterol
18	47	>90	Micro	Hypertension	Indapamide	Hypertensive nephropathy
19	73	82	Macro	Hypertension Hypercholesterolemia	Indapamide, Losartan, Atorvastatin	Minimal change disease and hypertensive nephropathy
20	58	85	Nil	Hypertension	Prazosin	Immunoglobulin A nephropathy

**Figure 1:**
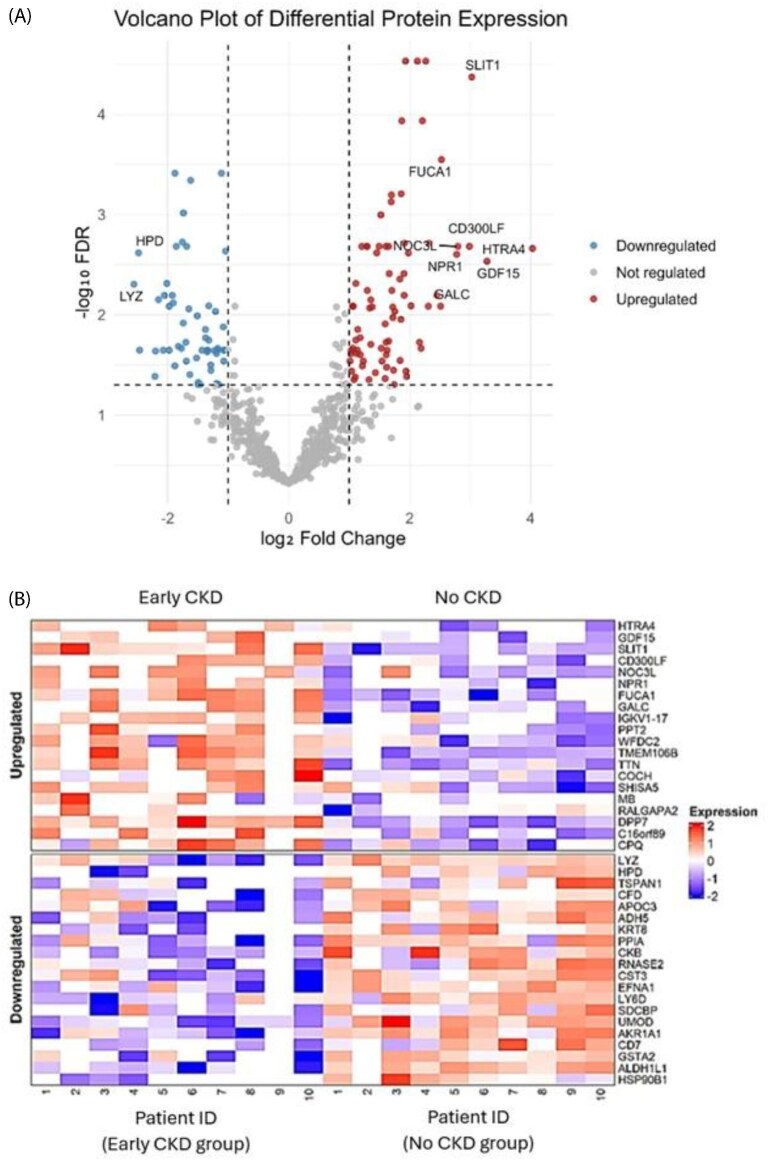
(**A**) Volcano plot of 759 proteins that were quantified, showing 237 proteins reaching the threshold of significant regulation (i.e. |log_2_FC|≥1, FDR < 0.1) (significantly upregulated proteins in red and significantly downregulated proteins in blue). (**B**) Heatmap displaying hierarchical clustering of the top 40 significantly regulated proteins (20 upregulated and 20 downregulated) between the early-stage CKD group and the no CKD group.

In total, 759 proteins were quantified through label-free proteomic methodologies, in which analysis of the human orthologue-mapped dataset through proteomic profiling detected 237 proteins with regulated expression where log₂ fold change was between 1 and −1, and false discovery rate was below 0.1. A volcano plot representing the findings from this analysis is presented, noting the most significantly upregulated and downregulated urinary proteins in early-stage non-diabetic CKD (Fig. 1A); namely, the most significantly upregulated urinary proteins in the presence of early-stage non-diabetic CKD included

HTRA4, GDF15, SLIT1, CD300LF, NOC3L, NPR1, FUCA1, and GALC and the most significantly downregulated urinary proteins included LYZ and HPD. A heatmap of the 20 most upregulated and 20 most downregulated proteins is also presented (Fig. 1B). Functional enrichment analysis validated the biological significance of these observed protein changes. Disruptions in lysosomal organization, extracellular matrix–receptor interaction, PI3K-Akt signalling pathways, and epithelial remodelling processes were the most significantly regulated pathways in early-stage CKD [4]. Kinase radar mapping analysis indicated significantly upregulated tyrosine (EPHA7, EPHB2, EPHB4, and DDR2) and serine/threonine kinases (MYLK, PAK6, and TTN) in early-stage CKD. These findings suggest intracellular signalling cascades are extensively perturbed, in line with the involvement of cytoskeletal remodelling, cell adhesion, and fibrotic signalling observed during CKD development [4, 5].

This study demonstrated various urinary proteins differed between the early-stage non-diabetic CKD and no CKD groups. The validity of using urinary proteomics as a biomarker tool in early-stage non-diabetic CKD is confirmed by reflecting similar molecular processes relating to the development of non-diabetic CKD as per findings from previously published studies [4, 5]. Further research is required to confirm the regulation of identified proteins in the kidneys of patients with early-stage non-diabetic CKD. Assessing the utility of urinary proteomic biomarkers for monitoring of treatment response in early-stage non-diabetic CKD is also anticipated.
